# Test–Retest Reliability of the Assessment of Fatty Liver Disease Using Low-Dose Computed Tomography in Cardiac Patients

**DOI:** 10.3389/fmed.2021.656658

**Published:** 2021-04-15

**Authors:** Antti Hokkanen, Hanna Hämäläinen, Tiina M. Laitinen, Tomi P. Laitinen

**Affiliations:** ^1^Department of Clinical Physiology and Nuclear Medicine, Kuopio University Hospital, Kuopio, Finland; ^2^Institute of Clinical Medicine, University of Eastern Finland, Kuopio, Finland

**Keywords:** computed tomography, non-alcoholic fatty liver disease, reliability, repeatability, test-retest, intraobserver, interobserver

## Abstract

Non-alcoholic fatty liver disease (NAFLD) is a common disorder that is associated with the risk of cardiovascular diseases. Therefore, its prevalence is high in patients with coronary artery disease. In myocardial perfusion imaging (MPI), low-dose computed tomography (CT) scans are used for attenuation correction in separate stress and rest studies. Here, the test–retest reliability of CT-based quantification of NAFLD was evaluated using these two CT scans. The study population consisted of 261 patients (156 men and 105 women, age 66 ± 10 years). Quantification of liver fat content was based on the radiodensity of the liver in Hounsfield units as well as in relation to corresponding values of the spleen. NAFLD was observed in 47 subjects (18%). CT quantification has good test–retest reliability in assessing NAFLD, with concordance correlation coefficient (CCC) ranging from 0.512 to 0.923, intraclass correlation coefficient (ICC) ranging from 0.513 to 0.923, and coefficient of variation ranging from 3.1 to 7.0%. Regarding the liver to spleen ratio, CCC for non-NAFLD patients and NAFLD patients was 0.552 and 0.911, respectively. For non-NAFLD patients ICC was 0.553 and NAFLD patients it was 0.913. The coefficient of variation for non-NAFLD and NAFLD patients was 4.9% and 3.1%, respectively. Our results suggest that low-dose CT is a feasible and well repeatable method but amount of liver fat contributes to repeatability. In NAFLD patients CCC and ICC were high reflecting excellent reliability, whereas in non-NAFLD patients test-retest reliability was moderate. Assessment of liver fat content can be used as additional information in studies where a CT scan has been done for other medical reasons, such as for low-dose attenuation correction CT along with MPI.

## Introduction

Non-alcoholic fatty liver disease (NAFLD) has become the most common cause of chronic liver disease in Western countries, with a global prevalence of 25.5% (1, 2). This has mainly been due to diet and lifestyle changes that have increased the prevalence of obesity and metabolic syndrome (MetS) (1, 3). In addition to NAFLD affecting the liver, it also increases the risk of type 2 diabetes mellitus, cardiovascular disease, and chronic kidney disease. Even though NAFLD is a global epidemic, the current gold standard of diagnosis remains liver biopsy, which is invasive and expensive (4). As NAFLD is asymptomatic for most patients, highly invasive methods cannot be extensively used for screening. Non-invasive methods include ultrasound (US), magnetic resonance imaging (MRI), and computed tomography (CT), all of which have their own flaws. The reliability of US depends greatly on the operator (5), MRI is expensive and has limited availability, and in CT the patient is exposed to radiation. However, CT-based NAFLD quantification is an attractive diagnostic tool. It is widely available, has better image quality than US, and is non-invasive. CT is already used in a wide range of investigations related to a variety of clinical problems, particularly in the thoracic and abdominal regions. The same images can also be used in the quantification of NAFLD, providing a cost-effective way of diagnosis (6). According to practical guidelines, NAFLD screening should be performed in people at high risk of cardiovascular diseases (3). In myocardial perfusion imaging (MPI) and single photon emission tomography (SPECT), low-dose CT scans are part of the imaging protocol and are used for attenuation correction. NAFLD can be assessed using the same CT scans, which can be used as additional information beyond the MPI results.

Testing reliability via test–retest studies requires two CT scans, which are commonly available in MPI studies (7). The protocol for MPI is to take two low-lose CT scans for attenuation correction, one for stress imaging and the other for rest imaging. Under these circumstances, we have been able to analyze two CT images for a large number of patients without additional exposure to radiation. The goal of this study was to evaluate the test–retest reliability of low-dose CT scans in the diagnosis of NAFLD and to calculate the limits of agreement for the method. To our knowledge, this is the first study evaluating the test–retest reliability of CT-based NAFLD quantification in a large sample of cardiac patients. We also assessed intra-observer and inter-observer variability for this method.

## Materials and Methods

### Study Population

The study population was formed from 586 patients who were referred for MPI during February 2010 and May 2011 at Kuopio University Hospital. We retrospectively analyzed 261 patients (156 men, 60%) who underwent both stress and rest phases of the study. The study protocol was approved by the Ethics Committee of the Northern Savo Hospital District.

Patient history (including previous diseases and laboratory values) was analyzed from the medical records. Due to retrospective design, we had no possibility to have complete data for all patients. Serum lipids were available for 227–234 subjects and plasma glucose for 231 subjects. Height and weight were measured (height to an accuracy of 1 cm and weight to an accuracy of 1 kg). Body mass index (BMI) was calculated as weight in kilograms divided by height in meters squared. Blood pressure was measured in the supine position from the brachial artery preceding the stress phase of the study. Blood pressure values were available for 255 subjects.

### Imaging Protocol

The MPI included SPECT with attenuation correction CT (SPECT/CT). Imaging was performed using a Philips Precedence hybrid camera, which has dual-headed gamma camera and a six-slice CT scanner (Philips Medical Systems, Bothell, WA). The stress phase included pharmacological provocation (adenosine or dobutamine) and when possible in combination with low-level exercise (starting with 20 W with increments of 20 W/min twice and the highest load was 60 W for 4 min) followed by SPECT/CT imaging 45 min after the provocation. The rest phase SPECT/CT imaging was performed on the same day at least 3 h after the first imaging. The attenuation correction CT was a low-dose CT (140 kV and 20–40 mAs). Both CT studies were performed with the same image parameters. The reconstruction was done for images of 5-mm slice thickness. This forms a test–retest setting for attenuation correction CTs.

### Measurements

**Figure 1:**
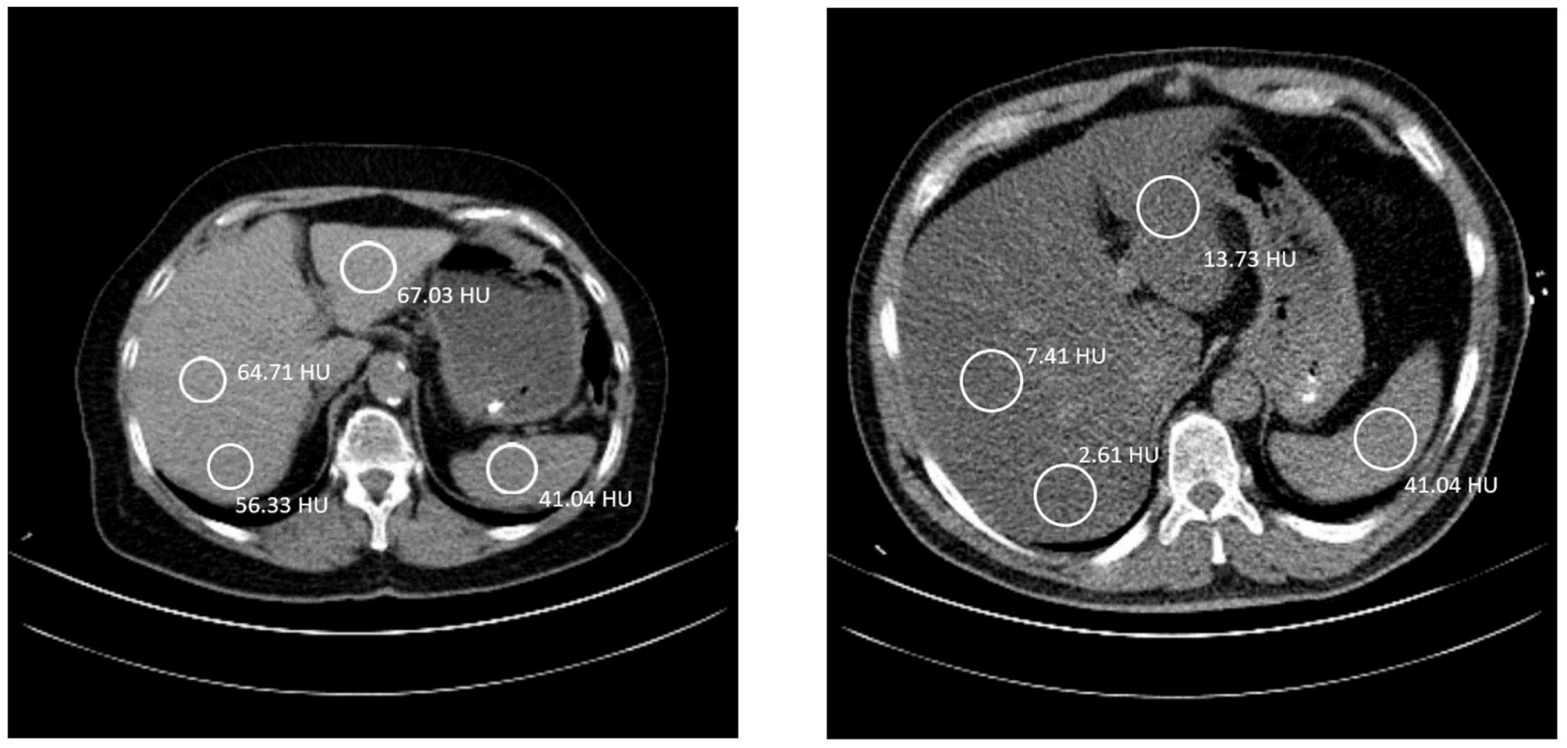
**Left**: A 70-year-old female, with liver to spleen ratio (L/S) of 1.15. The coloration of the liver is clearly brighter than the spleen. She has a BMI of 26.0 kg/m^2^ and has been diagnosed with hypertension, dyslipidemia, and a previous myocardial infarction. **Right**: A 50-year-old male, with L/S ratio of 0.12. The liver has a much darker color than the spleen, indicating a fatty liver. He has a BMI of 30.0 kg/m^2^ and has been diagnosed with type II diabetes mellitus, hypertension, and dyslipidemia. The patient also has less subcutaneous fat and more visceral fat. In both images the table is 40 cm wide. A good indication of the size difference between the two patients.

For each patient, Hounsfield unit (HU) values of four different regions of interest (ROIs) were measured according to Kerut et al. (8), one in the spleen and three in the liver. The spherical ROIs of the liver were spread out, one in the posterior part of the right lobe, one in the anterior part of the right lobe, and one in the left lobe (Figure 1). All ROIs were over 100 mm^2^ in size, and vasculature, cysts, and other heterogeneous areas were avoided. Measurements of HU values of selected ROIs were performed using IDS7 PACS (Sectra AB IDS7, 2019, version 21.1). The calculation of the liver to spleen ratio (L/S) was done by taking an average HU value of the two ROIs in the right lobe of the liver and dividing this value by the HU of the spleen, for example, L/S = [(49.3 + 51.3)/2]/43.9 = 1.15 (mean values from our results). The criterion for NAFLD was L/S < 1 based on average L/S of CT scan 1 and 2.

The first observer measured CT 1 scan and CT 2 scan for all 261 cases. For evaluation of inter-observer variability the second observer measured CT1 for 50 cases blinded to the initial results and repeated the measurements for evaluation of intra-observer variability.

### Statistical Methods

Two groups were formed according to the L/S. Patients with L/S < 1 were regarded as NAFLD patients (NAFLD+), and patients with L/S ≥ 1 were regarded as normal (NAFLD–). A Chi-square test was used to analyze the statistical significance of differences in the prevalence of obesity (BMI ≥ 30 kg/m^2^), type 2 diabetes, dyslipidemia, hypertension, and coronary artery disease between the groups. For continuous variables, a *T*-test was used to test the statistical significance of the difference between the two groups.

The concordance correlation coefficient (CCC) (9) and the intraclass correlation coefficient (ICC) (10) were used to assess the test–retest reliability between the measurements from CT scan 1 and CT scan 2. The coefficient of variation (CV%) was calculated according to Glüer et al. (11). The 95% limits of agreement (LoaA) were calculated according to the Bland–Altman method (12) as mean difference ± 1.96^*^standard deviation of the differences. Furthermore, to find out possible factors which may contribute to repeatability, a *T*-test was used to test statistical significance in absolute differences between measurements in CT 1 and CT 2 in which presence of NAFLD, obesity, dyslipidemia, type 2 diabetes, hypertension and coronary artery disease were used as grouping variables.

Inter-observer and intra-observer variability were expressed as CCC, ICC and LoA.

A *P*-value < 0.05 was considered statistically significant. SPSS software (IBM SPSS Statistics, 2013, version 22) was used to perform statistical analysis of the data.

## Results

Of the 261 patients enrolled in this study, 156 were men and 105 were women. The mean age was 66 years, with a range of 40–89 years. BMI for the pooled population was 29.5 kg/m^2^, with a range of 19.4–56.0 kg/m^2^. From the pooled population, 197 (75.5%) had been diagnosed with hypertension, 158 (60.5%) with dyslipidemia, 70 (26.8%) with type 2 diabetes, and 136 (52.1%) with coronary artery disease.

**Figure 2:**
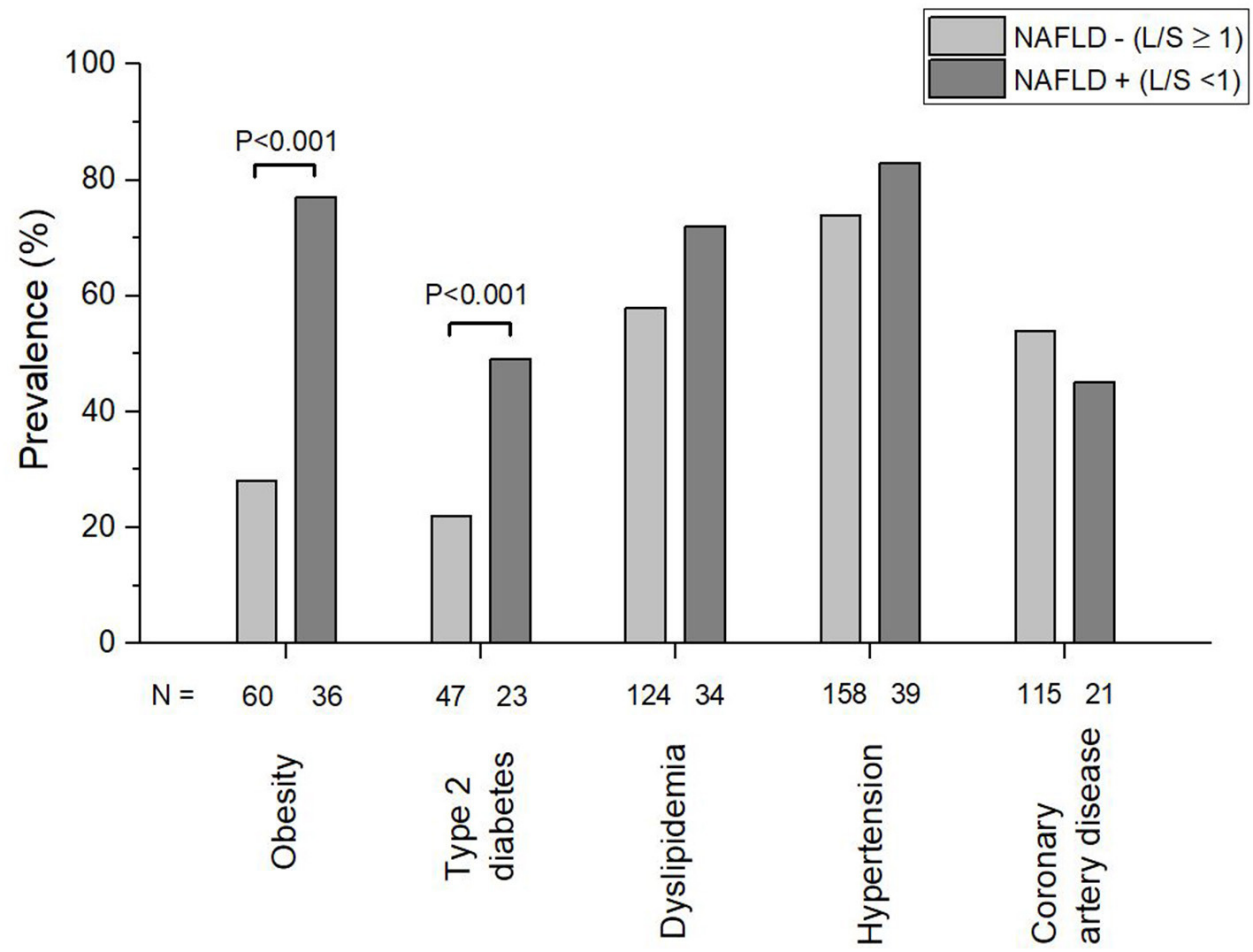
Prevalence of obesity, Type 2 diabetes, dyslipidemia, hypertension, and coronary artery disease in subjects with non-alcoholic fatty liver disease (NAFLD+) and without non-alcoholic fatty liver disease (NAFLD–). NAFLD was diagnosed using cut off value 1 for liver to spleen ratio (L/S) based on radiodensity values in low dose computed tomography scans.

The prevalence of NAFLD according to L/S < 1 in the study population was 18%. A diagnosis of NAFLD was not mentioned in the MPI reports in any of the cases. Between the groups, NAFLD+ showed a significantly higher prevalence in obesity (*P* < 0.001) and type 2 diabetes mellitus (*P* < 0.001) (Figure 2). The prevalence of dyslipidemia, hypertension and coronary artery disease was not significantly different between the two groups. In addition to the prevalence, the number of patients with obesity, type 2 diabetes mellitus, dyslipidemia, hypertension and coronary artery disease in each group is reported in Figure 2. Compared to subjects in the NAFLD– group, those in the NAFLD+ group were statistically significantly younger; had higher weight, BMI, serum triglyceride levels, and diastolic blood pressure; and had lower serum HDL cholesterol (Table 1). The sex distribution was comparable in the two groups.

**Table 1:** Clinical characteristics of the study population.

	**Pooled population (*n* = 261) Mean (SD)**	**NAFLD– (*n* = 214) Mean (SD)**	**NAFLD+ (*n* = 47) Mean (SD)**	***P*-value (NAFLD– vs. NAFLD+)**
Age (years)	66 (10)	67 (10)	63 (9)	0.004
Height (cm)	168 (10)	168 (10)	169 (9)	0.63
Weight (kg)	84 (19)	80 (16)	100 (24)	<0.001
BMI (kg/m^2^)	29.5 (6.3)	28.2 (5.1)	35.1 (8.0)	<0.001
Total cholesterol (mmol/l)	4.35 (1.09)	4.33 (1.04)	4.44 (1.31)	0.64
HDL cholesterol (mmol/l)	1.31 (0.40)	1.34 (0.41)	1.13 (0.29)	0.002
LDL cholesterol (mmol/l)	2.40 (0.93)	2.38 (0.90)	2.51 (1.04)	0.47
Triglycerides (mmol/l)	1.57 (0.98)	1.43 (0.76)	2.20 (1.50)	0.003
Glucose (mmol/l)	6.64 (1.81)	6.49 (1.64)	7.33 (2.31)	0.029
Systolic BP (supine, mmHg)	145 (23)	145 (23)	147 (21)	0.50
Diastolic BP (supine, mmHg)	76 (11)	75 (10)	80 (11)	0.004

*NAFLD–, subjects without non-alcoholic fatty liver disease; NAFLD+, subjects with non-alcoholic fatty liver disease; BMI, body mass index; HDL, high-density lipoprotein; LDL, low-density lipoprotein; BP, blood pressure*.

The mean radiodensity value according to average of CT scan 1 and 2 for the spleen ROI was 43.8 HU (SD 3.0, range 36.9–55.5 HU). The HU values for the ROIs in the liver right lobe were somewhat higher, with radiodensities of 48.9 HU (SD 9.2, range 5.3–66.3 HU) in the posterior ROI and 51.0 HU (SD 9.6, range 6.9–66.1 HU) in the anterior ROI. For the left lobe ROI, the equivalent values were 56.5 HU (SD 10.1, range 12.0–73.4 HU). The calculated mean L/S ratio for the pooled population was 1.15 (SD 0.23, range 0.14–1.57), while for the NAFLD- group the mean was 1.23 (SD 0.11, range 1.00–1.57) and for the NAFLD+ group the mean was 0.76 (SD 0.21, range 0.14–0.99). In Table 2 these values are reported separately in CT scan 1 and 2.

**Table 2:** Repeatability (test-retest) of parameters defined from the low-dose computed tomography.

	**CT scan 1 Mean (SD)**	**CT scan 2 Mean (SD)**	**CCC**	**ICC (95% CI)**	**CV%**	**95% limits of agreement**	**CT scan 2 - 1 difference Mean (SD)**
Spleen (HU)	43.9 (3.3)	43.7 (3.5)	0.512	0.513^*^ (0.418–0.597)	5.3	−6.9 to 6.4	−0.2 (3.4)
Liver right lobe (posterior) (HU)	49.3 (9.5)	48.4 (9.3)	0.920	0.920^*^ (0.895–0.939)	5.3	−8.1 to 6.3	−0.9 (3.7)
Liver right lobe (anterior) (HU)	51.3 (9.7)	50.6 (9.8)	0.923	0.923^*^ (0.902–0.940)	5.1	−8.1 to 6.7	−0.7 (3.8)
Liver left lobe (HU)	56.3 (10.4)	56.7 (10.3)	0.911	0.912^*^ (0.889–0.930)	5.3	−8.1 to 8.8	0.4 (4.3)
L/S (pooled population)	1.15 (0.24)	1.14 (0.24)	0.877	0.878^*^ (0.847–0.903)	7.0	−0.24 to 0.22	−0.01 (0.12)
L/S (NAFLD–)	1.24 (0.13)	1.23 (0.13)	0.552	0.553^*^ (0.453–0.640)	4.9	−0.25 to 0.23	−0.01 (0.12)
L/S (NAFLD+)	0.76 (0.22)	0.75 (0.21)	0.911	0.913^*^ (0.849–0.950)	3.1	−0.19 to 0.17	−0.01 (0.09)

CT, computed tomography; CCC, Concordance correlation coefficient; ICC, intraclass correlation coefficient; 95% CI, 95% confidence interval; CV%, coefficient of variation; HU, Hounsfield unit; L/S, average HU value of the liver right lobe/HU value of the spleen; NAFLD–, subjects without non-alcoholic fatty liver disease; NAFLD+, subjects with non-alcoholic fatty liver disease. Significances: ^*^P < 0.001.

The low dose CT showed moderate to excellent repeatability for all the measurements, with CCC ranging from 0.512 to 0.923. The CCC of L/S values for the NAFLD– group was lower than that for the NAFLD+ group at 0.552 compared to 0.911. Correspondingly ICC ranged from 0.513 to 0.923. For L/S it was 0.553 in NAFLD– group and 0.913 in NAFLD+ group. The CV% of L/S for the NAFLD– group was 4.9% and was 3.1% for the NAFLD+ group, both being significantly lower than the 7.0% for the pooled population. The LoA were −6.9 to 6.4 HU, −8.1 to 6.3 HU, −8.1 to 6.7 HU, and −8.1 to 8.8 HU for the spleen, the right lobe posterior ROI, the anterior ROI, and the left lobe, respectively. For the pooled population, the NAFLD– group, and the NAFLD+ group, the LoA of L/S ratios were −0.24 to 0.22, −0.25 to 0.23, and −0.19 to 0.17, respectively (Table 2).

**Figure 3:**
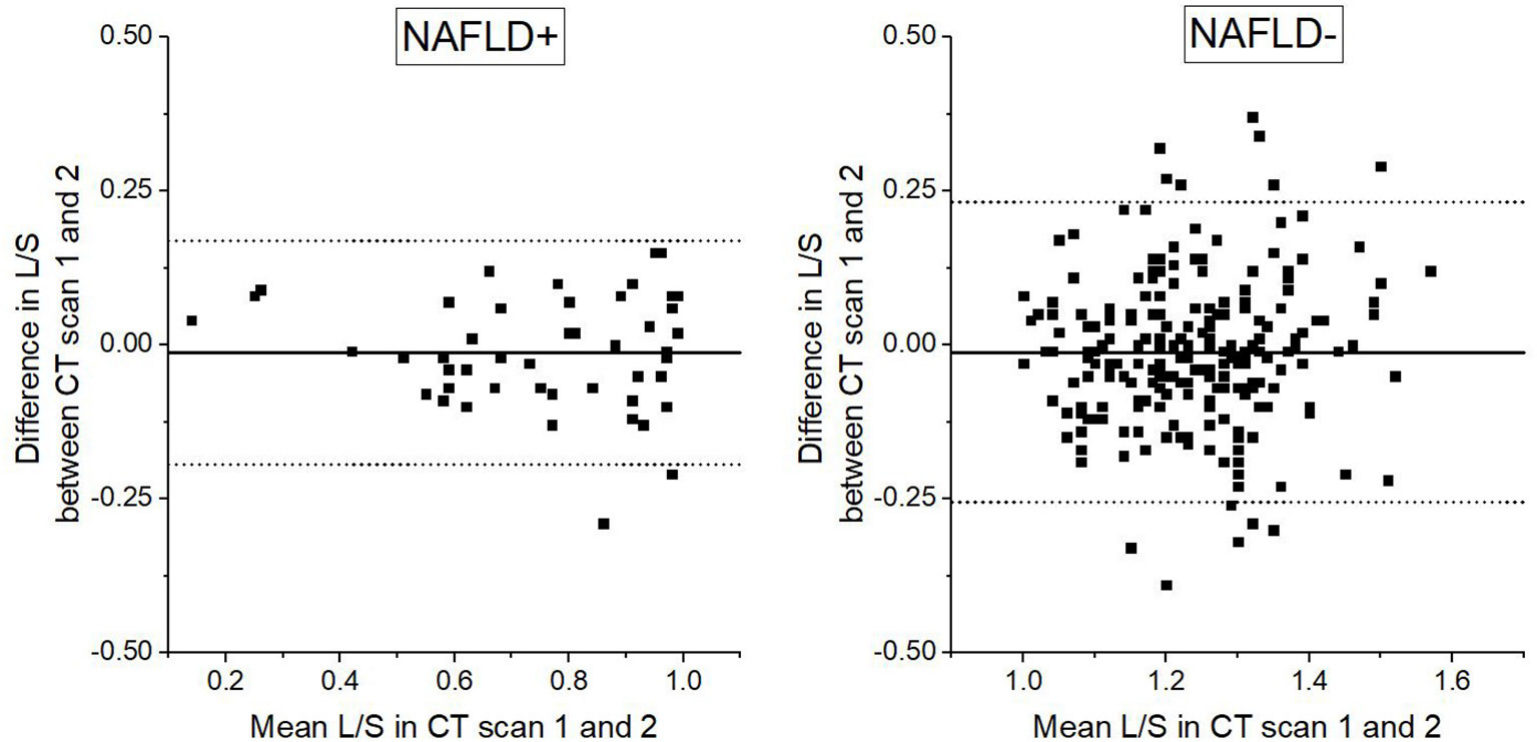
Bland-Altman plot demonstrating differences in liver to spleen ratio (L/S) based on radiodensity values in repeated low dose computed tomography scans in relation to mean of the two L/S assessments. Straight horizontal line represents mean difference, dotted horizontal lines represent upper and lower 95% limits of agreement. L/S cut of value of 1 is used in division of study population to subjects with non-alcoholic fatty liver disease (NAFLD+) and subjects without non-alcoholic fatty liver disease (NAFLD–).

Bland-Altman plot (Figure 3) shows that that the LoA are narrower in the NAFLD+ than in the NAFLD– group. NAFLD was the only factor of the clinical characteristics that contributed to the test–retest reliability. In the NAFLD+ absolute difference in L/S was 0.07 ± 0.05, whereas in the NAFLD- group it was 0.09 ± 0.07 (*P* = 0.037). The average absolute difference in L/S between CT scan 1 and CT scan 2 was comparable in subjects with and without type 2 diabetes (0.09 ± 0.07 vs. 0.09 ± 0.08, NS), in subjects with and without hypertension (0.09 ± 0.08 vs. 0.08 ± 0.07, NS), in subjects with and without coronary artery disease (0.09 ± 0.08 vs. 0.09 ± 0.07, NS), in subjects with and without dyslipidemia (0.09 ± 0.07 vs. 0.08 ± 0.08, NS), as well as in subjects with and without obesity (0.08 ± 0.07 vs. 0.09 ± 0.08, NS).

**Figure 4:**
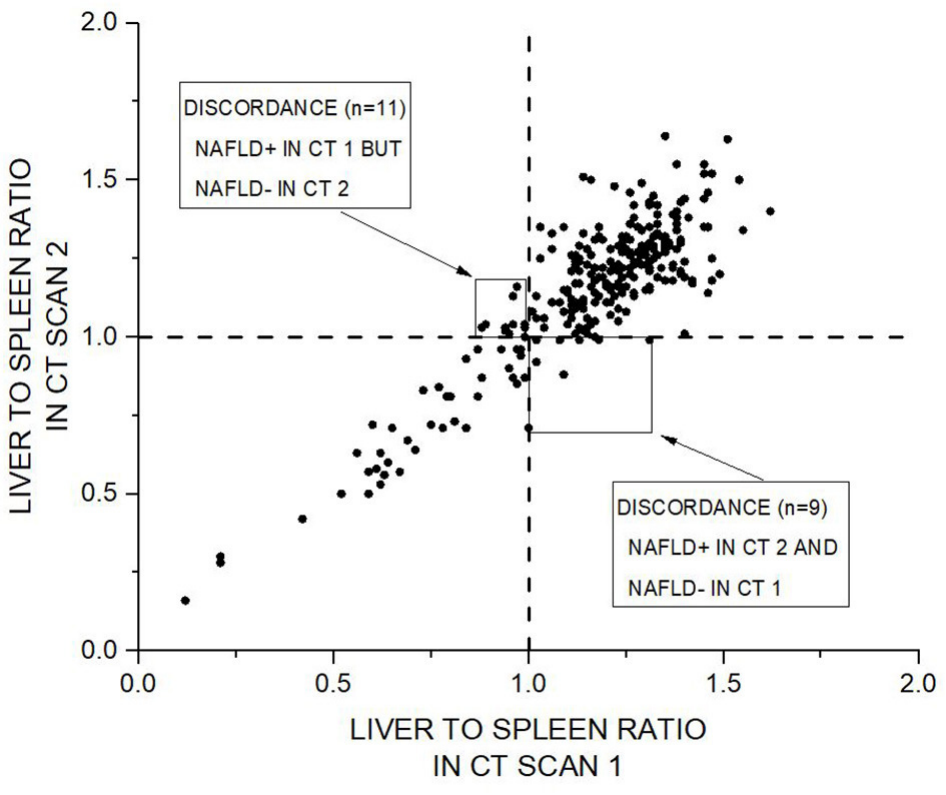
Scatter plot of liver to spleen ratio (L/S) in CT scan 1 vs. CT scan 2 demonstrating concordance for L/S based classification in repeated low dose computed tomography scans. Dashed vertical and horizontal lines represent L/S cut of value of 1, which is used in division of study population to subjects with non-alcoholic fatty liver disease (NAFLD+) and subjects without non-alcoholic fatty liver disease (NAFLD–). Totally 241 cases (92%) were classified concordantly in the CT scan 1 and CT scan 2. Twenty of cases (8%) were discordantly classified into NAFLD– or NAFLD+.

In patients with L/S below 1.00 in the CT-scan 1 (*n* = 47) CT scan 2 showed discordant result in 9 cases (19%) (Figure 4). However, totally 92% of cases were classified concordantly in the CT scan 1 and 2.

According to intra-observer variability analysis ICC was 0.805 for the spleen, and 0.966 for posterior part of the right lobe of liver, 0.967 for the anterior part of the right lobe, 0.957 for the left lobe and 0.949 for the L/S. In intra-observer LoA were −4.5 to 4.9 HU for the spleen, and −3.7 to 4.8 HU for the posterior part of the right lobe of liver, −3.7 to 5.3 HU for the anterior part of the right lobe, −4.9 to 5.5 HU for the left lobe and −0.14 to 0.16 for the L/S.

In the inter-observer variability analysis ICC was 0.710 for the spleen, and 0.928 for posterior part of the right lobe of liver, 0.924 for the anterior part of the right lobe, 0.899 for the left lobe and 0.906 for the L/S. In inter-observer LoA were −6.0 to 5.3 for the spleen, and −6.6 to 5.7 for the posterior part of the right lobe of liver, −4.2 to 8.0 for the anterior part of the right lobe, −8.4 to 7.8 for the left lobe and −0.16 to 0.22 for the L/S.

## Discussion

According to our analysis, CT has a good test–retest reliability in the assessment of NAFLD. This makes CT a reliable tool in NAFLD quantification. The small CV% (range 3.1–7.0%) also suggest that CT can be used in monitoring the progression of NAFLD. Clinical characteristics other than NAFLD, such as obesity, dyslipidemia, diabetes, hypertension and coronary artery disease, did not have a significant effect on the repeatability. Therefore, assessment of NAFLD using CT can be used widely in patients without clinical characteristics affecting the reliability. As the test–retest reliability was higher for patients with NAFLD than for patients without NAFLD, CT works well in monitoring patients who already have NAFLD. However, especially in borderline cases, diagnosis of NAFLD needs confirmation with alternative methods.

As we included subjects who were referred for MPI due to their clinical evaluation in relation to coronary artery disease and who already had all the CT images used in the study, we were able to analyze a large population (*n* = 261) without exposing study participants to excess radiation. Repeated low-dose CT scans had been taken of the patients due to the protocol used in MPI. This allowed us an opportunity to study the test–retest reliability. To our knowledge, no previous studies on CT-based test–retest reliability have been done due to the ethical problems of exposing patients to unnecessary radiation. Having a large population allows us to take into account possible confounding factors. Test –retest setting was obtained using the 1-day stress-rest protocol of MPI which includes two low-dose CT imaging primarily for attenuation correction for SPECT images. The interval between these was 3 h. Although the first imaging was done in stress phase we consider the two CT imaging situations to be well comparable regarding the assessment of liver fat content. The stress protocol which is used in myocardial perfusion imaging included adenosine infusion for 6 min and in most cases, additional low-level exercise on bicycle ergometer was used. The half time of adenosine is <10 s and due to the ultra-short half-life of adenosine its effects were fully expired before SPECT/CT imaging. Furthermore, exercise was mild and short lasting. Highest load was 60 W, which corresponds to walking outside for 4 min. After this stress procedure, subjects were sitting in the waiting room for 45 min. Because SPECT/CT was done 45 min after cessation of adenosine infusion and mild physical exercise, such post-stress situation is quite well comparable with the rest phase as regards to the abdominal CT analyses. This is supported by our finding that in stress and rest phases average differences (CT scan 2 – CT scan 1, Table 2, Figure 3) were near zero. Because there was only minor consistent difference, between the CT scan 1 and CT scan 2, these were true replicates of the measurements.

We decided to use a cut-off point of L/S < 1 to determine NAFLD based on a Japanese study, which showed sensitivity and specificity of 86.3 and 81.4%, respectively (13). Using reasonably small ROIs (over 100 mm^2^) allowed us to use homogenous liver tissue and to avoid the influence of other structures that would have affected the radiodensity values. By using this method, we found an NAFLD prevalence of 18%. This is almost the same as the prevalence of fatty liver in Finland of 18.5% (14) but is somewhat lower than the global prevalence of 25.5% and the previously reported European prevalence of 23.7% (2).

Compared to other test–retest repeatability studies on MRI- and US-based diagnosis, we see CT as a viable option. For MRI-based diagnosis (corrected T1 and MR elastography), the CV% ranged from 1.7 to 11% (15, 16) and for shear-wave ultrasonic elastography the CV% was 40% (16). Our calculations of the CV% for CT ranged between 3.1 and 7.0%. In comparing US and CT test–retest reliability, the ICC for US ranged from 0.58 to 0.82 (17) and for CT it ranged from 0.513 to 0.923.

Zeb et al. studied the inter- and intra-observer variability of CT-based NAFLD diagnosis using the same technique of calculating L/S ratios (18). Their inter-observer LoA for the liver and spleen were −5.63 to 5.25 and −5.68 to 7.15, respectively, and the intra-observer LoA for the liver and spleen were −2.79 to 2.51 and −3.94 to 4.42, respectively. In our study inter-observer and intra-observer variabilities were well in line with the previous study. As regards to two separate images (test – retest setting) LoA were somewhat higher at −6.30 to 8.10 and −6.41 to 6.86 for the liver and spleen, respectively, compared to LoA in intra-observer variability analysis. It is obvious that in repeated measurements variation results both from intra-observer variability in analyses as well as from physiological and technical differences in two separate imaging sessions. Interpretation of LoA should be based on a clinical context (12). Our results regarding LoA tell, which is the difference that will be exceeded of pairs of repeated measurements. This information can be useful in clinical situation when considering how reliable is CT based diagnosis and classification into NAFLD+ and NAFLD-, especially in borderline cases. This kind of uncertainty is also highlighted by our finding that in those patients with L/S below 1.00 in the CT-scan 1, the CT scan 2 showed discordant result in 9 cases (19%). On the other hand, totally 92% of cases were classified concordantly in the CT scan 1 and 2 (Figure 4).

We found that in test-retest analysis LoA were wider in the NAFLD– than in the NAFLD+ group. It can be speculated that repeatability is better in patients with NAFLD and the use of low dose CT is not as reliable in assessment of liver fat content in subjects without NAFLD. However, our observation may also be related to the phenomenon in which divergence depends on magnitude, i.e., when the differences increase in size proportionally to the size of measurement. In our study, for all parameters differences between CT scan 1 and CT scan 2 increased when magnitude of the measurements became higher. Thus, assumption of constant differences throughout the range was not met.

ICC and CCC were higher in measurements of the liver than in the spleen. Therefore, much of variability seems to be explained due to inconsistency in values of the spleen. We speculate that in the follow-up of liver disease progression, measurements of liver ROIs could be useful in addition to L/S.

We can also speculate that NAFLD would be a significant risk factor for cardiovascular diseases. As all the patients were admitted for MPI, they either had suspected or previously known coronary artery disease. Even though both groups had a similar prevalence of hypertension and coronary artery disease, for the NAFLD– group the mean age was 67 years, while for the NAFLD+ group the mean age was 63 years. This suggests that patients with NAFLD may have cardiovascular disease at a younger age than patients without NAFLD. The association between NAFLD and development of cardiovascular diseases was not analyzed thoroughly, and this needs attention in further studies.

From the results we can also see the association between NAFLD and MetS. The mean height was the same for both groups, but the mean weight was 20 kg higher for the NAFLD+ group. Consequently, the group also had an increased BMI of 35.1 kg/m^2^ compared to 28.2 kg/m^2^ for the NAFLD- group. Patients with an L/S ratio of <1 had a significantly higher prevalence of obesity, and type 2 diabetes mellitus, which are part of the International Diabetes Foundation MetS definition (19). In addition to a decreased liver radiodensity and a higher prevalence of some clinical problems, the NAFLD+ group also showed other characteristics of NAFLD and MetS, such as increased triglyceride levels, higher diastolic blood pressure, and decreased HDL levels compared to the NAFLD– group.

Even though the European Association of Nuclear Medicine (EANM) MPI guideline recommends screening images for extracardiac findings (7), none of the patients with an L/S ratio <1 had any mention of a fatty liver in their medical report. This highlights the under diagnosis of NAFLD in clinical practice. All these cases could have been diagnosed as a free by-product of the MPI SPECT/CT. As thoracic and abdominal CT scans are fairly common, if the image area contains the liver and spleen they could also be used to diagnose NAFLD as a by-product. For the patients with an indication of a fatty liver, further diagnosis should be performed. European practical guidelines highlight the need for NAFLD screening in people at high risk of cardiovascular diseases (3). Lifestyle changes toward a healthy diet, weight loss, and habitual physical activity are particularly emphasized in association with NAFLD (3).

Lack of information about alcohol consumption is an important limitation in the present study. Due to retrospective nature in our study design we were not able to collect exact such information and we had to rely on medical records in which this information is often inaccurate. We cannot exclude the possibility that some of our patients may have had fatty liver disease related to excessive alcohol consumption. However, the focus of this article is in repeatability of assessment of liver fat, and etiology of liver steatosis may not be crucial as regards to the main focus of our study. Additionally, none of the cases defined as NAFLD+ have been biopsy-proven. Comparative analysis with gold standard would have been valuable. Due to retrospective nature in our study design we were not able to choose to use reference methods. However, our study evaluating test – retest reliability gives a little different viewpoint to this issue. Variation due to technical or physiological reasons may partly limit the diagnostic performance, which however, seems to be good according to a previous study (13). Therefore, we focused on studying repeatability and reproducibility of the measurement to understand better the methodological variation.

In our study, the population has a high mean age of 66 years and a high prevalence of chronic diseases and therefore might not represent a general population. However, we want to encourage the assessment of NAFLD in patients undergoing MPI or in other situations where abdominal CT is available and NAFLD could be an additional finding. Furthermore, in clinical work screening for NAFLD can be performed using liver enzymes and/or US (3).

In conclusion, CT-based quantification of NAFLD has good test–retest reliability, and clinical characteristics other than NAFLD did not affect CT scan repeatability. Compared to MRI, CT shows similar reliability, but compared to US the test–retest reliability of CT seems to be even higher. Therefore, CT-based assessment of NAFLD and quantification could be used for diagnostic purposes. As the test–retest reliability was higher for patients with NAFLD than for patients without NAFLD, CT works well in monitoring patients who already have NAFLD. However, especially in borderline cases, diagnosis of NAFLD needs confirmation with alternative methods.

## Data Availability Statement

The datasets presented in this article are not readily available because permission to use this data is restricted by the General Data Protection Regulation (EU) 2016/679 and the Finnish authority (FINDATA) have defined the users and limited operating environment (THL/5431/14.02.00/2020). Output of Statistical analyses are available by request to the corresponding author (Tomi.Laitinen@kuh.fi).

## Ethics Statement

The studies involving human participants were reviewed and approved by The Ethics Committee of the Northern Savo Hospital District. Written informed consent for participation was not required for this study in accordance with the national legislation and the institutional requirements.

## Author Contributions

AH: methodology, investigation, and drafting the initial manuscript. HH: investigation and review or editing of the manuscript. TML: conceptualization/design, methodology, formal analysis, and review or editing of the manuscript. TPL: conceptualization/design, methodology, investigation, formal analysis, supervision/oversight, funding acquisition, resources, and review or editing of the manuscript. All authors contributed to the article and approved the submitted version.

## Conflict of Interest

The authors declare that the research was conducted in the absence of any commercial or financial relationships that could be construed as a potential conflict of interest.
